# From descriptive connectome to mechanistic connectome: Generative modeling in functional magnetic resonance imaging analysis

**DOI:** 10.3389/fnhum.2022.940842

**Published:** 2022-08-17

**Authors:** Guoshi Li, Pew-Thian Yap

**Affiliations:** ^1^Department of Radiology, University of North Carolina, Chapel Hill, NC, United States; ^2^Biomedical Research Imaging Center, University of North Carolina, Chapel Hill, NC, United States

**Keywords:** computational modeling, brain network, dynamical causal model, biophysical network model, Dynamic Neural Model, neural mass model, connectome, fMRI

## Abstract

As a newly emerging field, connectomics has greatly advanced our understanding of the wiring diagram and organizational features of the human brain. Generative modeling-based connectome analysis, in particular, plays a vital role in deciphering the neural mechanisms of cognitive functions in health and dysfunction in diseases. Here we review the foundation and development of major generative modeling approaches for functional magnetic resonance imaging (fMRI) and survey their applications to cognitive or clinical neuroscience problems. We argue that conventional structural and functional connectivity (FC) analysis alone is not sufficient to reveal the complex circuit interactions underlying observed neuroimaging data and should be supplemented with generative modeling-based effective connectivity and simulation, a fruitful practice that we term “mechanistic connectome.” The transformation from descriptive connectome to mechanistic connectome will open up promising avenues to gain mechanistic insights into the delicate operating principles of the human brain and their potential impairments in diseases, which facilitates the development of effective personalized treatments to curb neurological and psychiatric disorders.

## Introduction

The human brain is a fascinating machine in which the interactions of vast numbers of distributed circuits and networks give rise to complex cognitive functions such as perception, attention, decision making and memory. Delineating the brain’s anatomical wiring diagram and its functional operation principles constitutes an important first step to decipher the underlying mechanisms of cognition. Connectomics is a newly developed field dedicated to providing a complete map of neuronal connections in the brain (Sporns et al., 2005). Fueled by the rapid advances of non-invasive neuroimaging techniques, connectomics has become one of the most vibrant disciplines in neuroscience (Craddock et al., 2013). As a major international neuroscience initiative, the Human Connectome Project (HCP) aims to build a network map of neural systems by systematically characterizing structural and functional connectivity (FC) of the human brain (Van Essen and Glasser, 2016). The project has made ground-breaking achievements in both method development and scientific discoveries, which acquired and analyzed a wealth of multimodal MRI and magnetoencephalography (MEG) data of unprecedented quality for further exploration of brain cognitive functions (Elam et al., 2021). Connectomics is not only important for studying normal brain functions but also highly promising to understand the pathological basis of neurological and psychiatric disorders for better diagnosis and treatments (Deco and Kringelbach, 2014).

As the essential components of human connectomics, structural and functional connectomes are driven by two major imaging modalities, diffusion MRI and functional MRI, respectively. Diffusion MRI is a method based on measuring the random Brownian motion of water molecules within the white matter (Baliyan et al., 2016), which can be used to infer long-distance whiter mater tracts (tractography) that connect distant brain regions informing structural connectivity (SC) (Van Essen and Glasser, 2016). Such structural information describes the physical substrate underlying brain functions. On the other hand, functional MRI (fMRI) is a class of imaging methods designed to measure regional, time-varying changes in brain metabolism in response to either task stimuli (task-fMRI) or spontaneous modulation of neural process (resting-state fMRI) (Glover, 2011). The most common form of fMRI employs blood oxygen level-dependent (BOLD) contrast imaging which measures variations in deoxyhemoglobin concentrations, an indirect measure of neuronal activity (Soares et al., 2016; Chow et al., 2017). BOLD-fMRI has been widely used for large-scale brain mapping (Gore, 2003; Just and Varma, 2007; Glover, 2011; Power et al., 2014) and the fMRI data is predominantly analyzed using FC, which is defined as the statistical dependencies among fluctuating BOLD timeseries from distributed brain regions (Friston, 2011). FC can be computed using either simple correlation analysis (e.g., Pearson’s correlation) or more sophisticated statistical methods such as mutual information, independent component analysis and Hidden Markov models (Karahanoglu and Van De Ville, 2017; Vidaurre et al., 2017; Bolton et al., 2018).

Despite the great success and widespread use of SC and FC in characterizing the organizational principles of large-scale brain networks, their application to fundamental cognitive neuroscience problems and clinical treatments is still limited due to several notable limitations. *First of all*, such macroscopic connectivity analysis is largely descriptive and superficial (Stephan et al., 2015; Braun et al., 2018), thus unable to offer a mechanistic account of the neural process underlying cognitive function or dysfunction. *Second*, both SC and FC are undirected and unsigned. Consequently, they cannot model the inherent asymmetry observed in both anatomical and functional connections (Felleman and Van Essen, 1991; Frässle et al., 2016). In addition, the unsigned connections imply that SC and FC are not able to infer excitatory or inhibitory coupling strengths for excitation-inhibition (E-I) balance estimation. This disadvantage is non-trivial because E-I balance plays an important role in neural coding, synaptic plasticity, and neurogenesis (Froemke, 2015; Deneve and Machens, 2016; Lopatina et al., 2019). *Lastly*, neither SC or FC captures intrinsic or intra-regional connections. As cortical neurons are more subject to the influence of short-range (local) than long-range (inter-regional) connections (Unal et al., 2013), an alternative connectivity measure or modeling approach is needed to account for intrinsic neural interactions for a more comprehensive characterization of connectome.

To overcome the limitations of SC and FC, Friston and colleagues introduced the concept of effective connectivity (EC), which is defined as the directed causal influence among neuronal populations (Friston et al., 2003; Friston, 2011). Different from SC and FC, EC builds on a generative model of neural interactions and corresponds to the coupling strengths of the neuronal model estimated from the observed neuroimaging data (Friston et al., 2003; Friston, 2011). As the generative model describes how the latent (hidden) neuronal states and their interactions give rise to the observed BOLD measurements (for fMRI), EC can potentially provide mechanistic neuronal accounts of the fMRI data under both normal cognitive process and abnormal disease state. In addition, EC is directed and signed, allowing estimation of excitatory and inhibitory coupling strengths for E-I balance inference. Furthermore, by incorporating more fine-grained microscopic or mesoscopic neuronal models, intra-regional connection strengths can also be estimated. Thus, effective connectivity based on generative modeling provides a highly significant complementary approach to conventional SC and FC analysis, which enables deeper mechanistic understanding of functional connectome.

This review paper attempts to give a concise overview of existing generative modeling approaches in fMRI analysis and how they contribute to the emerging and rapidly growing field of human connectomics. We first define generative modeling in a general sense, discuss their important roles in neuroscience, and introduce three major generative modeling frameworks in fMRI connectome analysis. Next we review the state-of-the-art of each of the three major generative modeling approaches and sample their applications to cognitive or clinical neuroscience problems. Lastly, we summarize the developments and achievements of generative models in fMRI connectome analysis and discuss future research directions for this dynamic and promising field in neuroscience.

## Generative modeling of human connectomics

Generative models are computational models that are built to simulate the response profile of a complex system based on the physical mechanisms of interactive components (Li and Cleland, 2018). Such models are designed to generate proper values for all embedded variables with interpretable physical meaning rather than deriving certain properties from a “black box” to study certain higher-level phenomena (Li and Cleland, 2018). Generative modeling has many useful applications, such as interpreting the underlying mechanisms of emergent properties in complex systems, testing the sufficiency of working hypothesis, and making testable predictions to guide experimental design. Since the introduction of the classical Hodgkin and Huxley model (Hodgkin and Huxley, 1952), generative modeling has become a core component in computational neuroscience. Traditional generative neuronal models (based on non-human data) have been focused on how collective properties of neurons, synapses and structures give rise to network activity and dynamics to sustain neural circuit functions (e.g., Hasselmo et al., 1995; Durstewitz et al., 2000; Li et al., 2009; Markram et al., 2015; Hass et al., 2016). Such detailed biophysical neuronal models involve collecting sufficient anatomical and electrophysiological data at the cellular and synaptic levels mostly from non-human subjects. Generative models can then be constructed by integrating the anatomical and physiological data with known neuronal biophysics within a microscale circuit. A notable example for such detailed modeling is the Blue Brain project, an international initiative that aims to create a digital reconstruction of the mouse brain to identify the fundamental principles of brain structure and function (Markram, 2006).

The generative models for human connectomics have several distinct features compared with detailed microcircuit models for non-human data. *First*, they focus on the estimation of effective connectivity or coupling strengths among neural populations. *Second*, they are driven and constrained by neuroimaging data such as diffusion MRI, fMRI and MEG, and seek to reveal the neuronal mechanisms underlying the observed data. *Third*, they generally apply population-level (neural mass) models to describe network dynamics as such dynamic models relate more closely to macroscopic neuroimaging data by describing the collective activity of neuronal populations (Breakspear, 2017). *Lastly*, generative models for connectomics commonly involve an optimization strategy to estimate the coupling strengths among neural populations based on neuroimaging data. This contrasts with detailed microcircuit neuronal models that usually infer model parameters from anatomical and electrophysiological data. Thus, generative models for connectomics consist of three essential components: (1) a neuronal model that generates population-level neural activity; (2) an observation model that transforms the neural activity to simulated neuroimaging data; and (3) an optimization scheme to estimate connection parameters.

Depending on the differences in neuronal models, the scope of parameter estimation and the optimization scheme, existing generative models for connectomics can be broadly classified into three major types: (1) Dynamic Causal Model (DCM); (2) Biophysical Network Model (BNM); and (3) Dynamic Neural Model (DNM) with direct parameterization. Below we review the foundation and development of these three types of generative models and survey their applications to cognitive or clinical neuroscience.

## Dynamic causal modeling

### Overview

**FIGURE 1:**
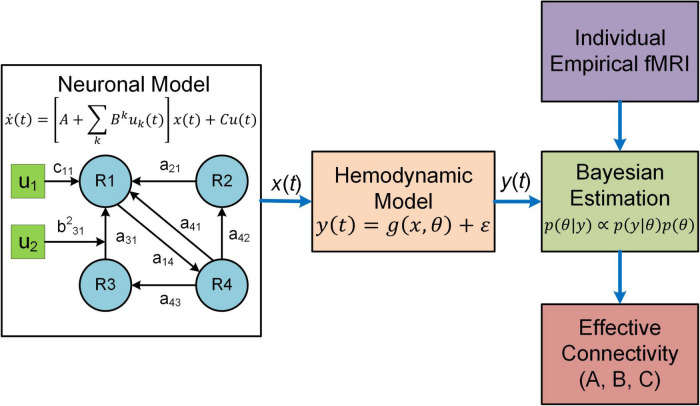
Overview of DCM. In DCM, neural interactions among different brain regions (R1, R2, etc.) are described by a bilinear model. Effective connectivity includes both a baseline (*a*_21_ *a*_31_, etc.) and a modulatory component (e.g., *b*^2^_31_) due to exogenous experimental inputs (*u*_1_, *u*_2_), whereas the matrix *C* represents the influence of external inputs on neural activity. The regional neural activity [*x*(*t*)] is converted to BOLD response [*y*(*t*)] *via* a biophysical hemodynamic model. With individual empirical fMRI data, DCM estimates effective connectivity (the matrices *A* and *B*) as well as the matrix *C* using Bayesian estimation technique.

Dynamic causal modeling (DCM) is a predominant analysis framework to infer effectivity connectivity of individual connections at single subject level using standard Variational Laplace procedures (Friston et al., 2003, 2007). A typical DCM contains a forward (generative) model that describes the dynamics of interacting neuronal populations and a measurement model that converts the neuronal activity into measurable observations such as fMRI, MEG and electroencephalogram (EEG) (Figure 1). During model inference, DCM simulates the BOLD responses for models with different configurations of connectivity and determines the model that best characterizes the empirical fMRI data. DCM adopts a two-stage process to estimate EC (Zeidman et al., 2019a,b). The first stage is Bayesian model inversion (estimation), a process that finds the parameters which provide the best trade-off between model accuracy (how good the model fits the data) and model complexity (how far the parameters need to deviate from their prior values to fit the data); such trade-off is quantified as model evidence. The second stage is termed Bayesian model comparison where models with different network connectivity are compared based on evidence either at the single-subject or group level. For detailed procedures about the two-stage process, see two recent tutorial papers on DCM (Zeidman et al., 2019a,b).

The original deterministic DCM applies to task-fMRI only and is restricted to relatively small networks (< 10 brain regions) due to computational burden (Daunizeau et al., 2011). To account for resting-state activity, two variations of DCM, stochastic DCM (Li et al., 2011) and spectral DCM (Friston et al., 2014), have been developed. Stochastic DCM estimates EC as well as random fluctuations on both neural states and measurements, which is computationally intensive and thus can handle only a few numbers of brain regions. By comparison, spectral DCM estimates the parameters of cross-spectral density of neuronal fluctuations instead of time-varying fluctuations in neuronal states, which is much more stable and computationally efficient. By using FC as prior constraints, the computational efficiency of spectral DCM is further improved, enabling modeling of relatively large networks (36 nodes, Razi et al., 2017).

The original DCM is limited to modeling one neural population for each brain region, which is later extended to two-state DCM to account for the intra-regional interactions between excitatory and inhibitory neural populations (Marreiros et al., 2008). Despite the added biological realism, the two-state DCM is still a linear model, and consequently may not accurately represent the brain neural dynamics in the long term (Singh et al., 2020). A major advancement to DCM for fMRI was introduced recently by replacing the bilinear model with a neural mass model (NMM) of the canonical microcircuit (Friston et al., 2019). Specifically, four neural populations are modeled in each brain region coupled with both inter- and intra- laminar connections of the cortical microcircuitry; each neural population is further represented by two hidden states whose dynamics are governed by second-order differential equations. By incorporating a sophisticated and physiologically informed NMM, DCM for fMRI parallels the development of DCM for electrophysiological data (Moran et al., 2011; Friston et al., 2012), which is able to provide deeper mechanistic insights for observed fMRI data. It should be noted that both the earlier two-state DCM or the latest NMM-based DCM are designed for task-fMRI, although one could potentially apply them to the resting-state condition by modeling the exogenous inputs as Fourier series (Di and Biswal, 2014).

Despite the significant technical advance of DCM in both model scope (i.e., extension to resting-state fMRI) and model complexity (i.e., use of sophisticated NMM), computational efficiency remains a major limitation. Even for the most efficient spectral DCM, inversion of a medium size network with 36 nodes takes about 20–40 h (Razi et al., 2017), limiting its application to whole-brain network and big dataset. To address this limitation, a novel variant of DCM (i.e., regression DCM) has been developed (Frässle et al., 2017, 2018, 2021a,b). Regression DCM (rDCM) converts the linear DCM equations from the time domain to the frequency domain, enabling efficient solution of differential equations using Fourier transformation. Together with other assumptions such as fixed hemodynamic response function, rDCM treats model inversion in DCM as a special case of Bayesian linear regression problem. These technical innovations have equipped rDCM with extremely high computational efficiency, which requires just a few seconds to estimate EC of a whole-brain network with 66 nodes (Frässle et al., 2017). Later technical improvements such as sparsity constraints in rDCM enable EC estimation of larger brain-wide networks with denser connections (> 200 regions, > 40,000 connections) within only a few minutes (Frässle et al., 2018, 2021b). Together with the latest extension to resting-state fMRI (Frässle et al., 2021a), rDCM opens promising new opportunities for human connectomics. Nevertheless, current rDCM is inherently linear and has not incorporated more realistic generative NMMs, which hinders its application to fundamental neuroscience problems. Other variants of DCM include sparse DCM (Prando et al., 2019) and DCM with Wilson-Cowan-based neuronal equations (Sadeghi et al., 2020).

### The neural and measurement models

The original DCM uses a bilinear state space model (Friston et al., 2003):


(1) $$\dot{x}\,\left(t\right)=\left[A+\sum_{\text{k}}B^{\text{k}}\,u_{\text{k}}\,\left(t\right)\right]\,x\,\left(t\right)+C\,u\,\left(t\right)$$

where *x*(*t*) denotes the hidden neuronal states for multiple brain regions, *u*(*t*) represents exogenous experimental inputs and the matrix *C* models the influence of external inputs on neuronal activity. *A* is the baseline effective connectivity and *B*^k^ represents the modulation on effective connectivity due to the input *u*_k_(*t*). DCM estimates the parameters *A*, *B*^k^ and *C* based on fMRI data. For the generative neural models of other variants of DCM, refer to related publications (Marreiros et al., 2008; Li et al., 2011; Friston et al., 2014, 2019; Frässle et al., 2017, 2018).

DCM employs a biophysical hemodynamic model to translate the regional neural activity *x*_*i*_(*t*) to observed BOLD response *y*_i_(*t*) (Friston et al., 2003). Specifically, for each region *i*, the fluctuating neuronal activity *x*_i_(*t*) leads to a vasodilatory signal *s*_i_(*t*) which is subject to self-regulation. The vasodilatory signal induces change in the blood flow *f*_i_(*t*) resulting in subsequent change in blood volume *v*_i_(*t*) and deoxyhemoglobin content *q*_i_(*t*). The hemodynamic model equations are given as follows (Friston et al., 2003):


(2) $$\frac{d\,s_{\text{i}}\,\left(t\right)}{d\,t}=x_{\text{i}}\,\left(t\right)-\kappa \,s_{\text{i}}\,\left(t\right)-\gamma \,\left(f_{\text{i}}\,\left(t\right)-1\right)$$

(3) $$\frac{d\,f_{\text{i}}\,\left(t\right)}{d\,t}=s_{\text{i}}\,\left(t\right)$$

(4) $$\tau \,\frac{d\,v_{\text{i}}\,\left(t\right)}{d\,t}=f_{\text{i}}\,\left(t\right)-v_{\text{i}}^{\frac{1}{\alpha }}\,\left(t\right)$$

(5) $$\tau \,\frac{d\,q_{\text{i}}\,\left(t\right)}{d\,t}=\frac{f_{\text{i}}\,\left(t\right)}{\rho }\,\left[1-{\left(1-\rho \right)}^{1/f\,\left(t\right)}\right]-\frac{q_{\text{i}}\,\left(t\right)}{v_{\text{i}}\,\left(t\right)}\,v_{\text{i}}^{\frac{1}{\alpha }}\,\left(t\right)$$

where κ is the rate of decay, γ is the rate of flow-dependent elimination, τ is the hemodynamic transit time, α is the Grubb’s exponent and ρ is the resting oxygen extraction fraction. The predicted BOLD response is calculated as a static non-linear function of blood volume and deoxyhemoglobin content that depends on the relative contribution of intravascular and extravascular components:


(6) $$y_{\text{i}}\,\left(t\right)=v_{0}\,\left(k_{1}\,\left(1-q_{\text{i}}\,\left(t\right)\right)+k_{2}\,\left(1-q_{\text{i}}\,\left(t\right)/v_{\text{i}}\,\left(t\right)\right)+k_{3}\,\left(1-v_{\text{i}}\,\left(t\right)\right)\right)$$

where *v*_*0*_ is the resting blood volume fraction, and *k*_*1*_, *k*_*2*_ and *k*_*3*_ are the intravascular, concentration and extravascular coefficients, respectively.

### Development and implementation of dynamic causal models

Development of DCM models involves several major steps: **(1)** experimental design and hypothesis formulation; **(2)** selection of regions and extraction of fMRI-BOLD timeseries; **(3)** selection of DCM model depending on the fMRI modality (task or resting-state), network size and the problem of interest (i.e., one state, two-state or NMM-based DCM); **(4)** specification of the neural model including network connectivity and experimental inputs; **(5)** model estimation at subject-level; and **(6)** group-level analysis with Parametric Empirical Bayes (PEB; Friston et al., 2016). DCM is implemented using the SPM software package^1^ running under MATLAB. The detailed implementation procedures can be found in two recent DCM guide papers (Zeidman et al., 2019a,b).

### Applications to cognitive neuroscience

As the predominant analysis method to compute EC, DCM has been used extensively to study cognitive problems in neuroscience. Below, we review a few examples from the huge literature that showcase the applications of DCM to understand cognitive processes including attention, perception, emotion and decision making. Cognitive information processing is regulated by two fundamental principles including functional separation and functional integration (Sporns, 2013; Deco et al., 2015; Wang et al., 2021). To investigate the basic connectivity architecture of neural structures in goal-directed attentional processing, Brázdil et al. (2007) conducted an event-related fMRI study employing the visual oddball task, one of the most widely used experimental paradigms in cognitive neuroscience. The deterministic DCM and bayes factors were applied to infer the coupling strengths among different brain regions and the parameters embodying the influence of experimental inputs on connectivity, and to compare competing neurophysiological models with different intrinsic connectivity. The study revealed that a bidirectional frontoparietal information flow exists [from intraparietal sulcus (IPS) to prefrontal cortex (PFC) and from anterior cingulate cortex (ACC) to PFC and the IPS] during target stimulus processing, which may indicate simultaneous activation of two distinct attentional neural systems. Compared with exteroceptive attention (i.e., inputs from external environment), the neural mechanism of interoceptive attention (i.e., the awareness and conscious focus toward physiological signals arising from the body) is much less known. To evaluate the functional role of the anterior insular cortex (AIC) in interoceptive attention, Wang et al. (2019b) applied a novel cognitive task that directed attention toward breathing rhythm and utilized DCM analysis of fMRI data to explain the potential mechanisms of interaction between AIC and other brain regions. After model inversion, random-effects Bayesian Model Selection (BMS; Stephan et al., 2009) was applied to determine the best model from 52 candidate models based on the observed data from all participants. The authors reported that interoceptive attention was associated with elevated AIC activation, increased coupling strength between AIC and somatosensory areas, and weaker coupling between the AIC and visual sensory areas. Notably, the differences in individual interoceptive accuracy can be predicted by AIC activation, suggesting the essential role of AIC in interoceptive attention.

DCM has also been applied to study perceptual learning, a process of improved perceptual performance after intensive training. To examine how perceptual learning modulates the responses of decision making-related regions, Jia et al. (2018) combined psychophysics, fMRI and model-based approach, and trained participants on a motion direction discrimination task. DCM models were constructed to examine whether learning changes the EC among V3A, middle temporal area (MT), intraparietal sulcus (IPS), frontal eye filed (FEF) and ventral premotor cortex (PMv). The network receives external motion inputs from both V3A and MT with motion direction (trained vs. untrained). BMS with random effect analysis (Stephan et al., 2009) was applied to test nine candidate models with different modulation assumptions. Results indicated that learning strengthened the EC on the feedforward connections from V3A to PMv and from IPS to FEF, suggesting that perceptual learning leads to decision refinement. In a recent study, Lumaca et al. (2021) employed DCM in conjunction with PEB analysis to identify modulation of brain EC during perceptual learning of complex tone patterns based on fMRI of a complex oddball paradigm. The authors found that errant responses were associated with excitation increase within the left Heschl’s gyrus (HG) and left-lateralized increase in feedforward EC from the HG to the planum temporale (PT), which suggests that the prediction errors of complex auditory learning are encoded by connectivity changes in the feedforward and intrinsic pathways within the superior temporal gyrus.

In addition to task-fMRI, resting-state fMRI has been analyzed by DCM models to study the neural substrate of cognition. Esménio et al. (2019) combined functional and effective connectivity analysis to characterize the neurofunctional architecture of empathy in the default mode network (DMN). They performed resting-state fMRI scan on 42 participants who completed a questionnaire of dyadic empathy. Using spectral DCM on resting-state fMRI data, they observed that subjects with higher scores in empathy showed stronger EC from the posterior cingulate cortex (PCC) to bilateral inferior parietal lobule (IPL), and from the right IPL to the medial prefrontal cortex (mPFC). Such findings support the hypothesis that individual difference in self-perceived empathy is mediated by systematic variations in effective connectivity within the DMN, which underlie differences in FC.

## Biophysical network model

### Overview

**FIGURE 2:**
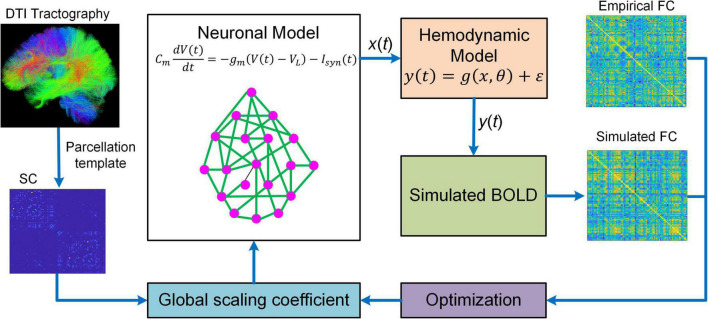
Overview of BNM. BNM is large-scale whole brain network model containing up to hundreds of network nodes (i.e., brain regions). For a parcellation template, the connectivity of different brain regions is determined by structural connectivity (SC) informed from diffusion tensor imaging (DTI) tractography. The SC is scaled by a global coefficient to model synaptic efficiency or strengths among remote neural populations. The neuronal dynamics of each network node is described by first-order differential equation modeling the membrane potential of individual neurons. The regional neural activity [*x*(*t*)] is transformed into BOLD signal [*y*(*t)*] *via* a hemodynamic model from which simulated functional connectivity (FC) is computed. BNM fits simulated FC to empirical FC by optimizing the global scaling coefficient. After fitting, BNM can be applied to simulate fMRI response and study the relationship between SC and FC.

Besides DCM, BNM is another popular generative model that has been proved to be useful in studying fMRI connectome. BNM for fMRI is a modeling framework that incorporates structural and physiological properties of brain networks, represents each network node with populations of neurons, and connects distinct nodes with long-range fibers estimated from diffusion MRI data to simulate fMRI responses (Figure 2; Honey et al., 2007, 2009; Deco and Jirsa, 2012; Deco et al., 2013a,b). BNM is typically large-scale whole brain network model comprising of up to 1,000 network nodes (Honey et al., 2007, 2009; Deco et al., 2013a,b; Sanz-Leon et al., 2015). There are two major approaches to model the neuronal dynamics of local network node. The first approach is “direct simulations” where a large number of individual neurons linked by local synaptic rules are simulated (Stephan et al., 2015). This approach is similar to detailed biophysical microcircuit modeling, though in BNM individual neurons are usually modeled by simplified spiking models instead of full-scale conductance-based compartmental models (i.e., Hodgkin and Huxley type model). The major drawbacks of “direct simulations” include heavy computational burden and large number of loosely constrained model parameters often requiring inference from animal electrophysiological data, which makes systematic exploration of parameter space and conclusive analysis infeasible (Stephan et al., 2015). An alternative method is to represent each network node with a neural mass or mean-field model of local neuronal populations, often referred to as a mean-field reduction approach (Deco and Jirsa, 2012; Deco et al., 2013b). Due to the tractability and balance between biophysical realism and model complexity, the mean-field modeling approach has become the mainstream of current BNMs of neuroimaging data.

Despite increased tractability of the mean-field reduction approach, BNM is still highly complex because of large network size and inherent non-linearity. As a result, it is extremely challenging to estimate the coupling strengths of all individual connections. Consequently, BNMs have been focusing on simulating fMRI data using SC as a proxy for synaptic weights (Honey et al., 2007, 2009; Deco et al., 2013a; Jirsa et al., 2017), estimating only one single global scaling coefficient for all inter-regional connections (Deco et al., 2013b; Wang et al., 2019a), or estimating a small subset of connection parameters typically at group-average level (Deco et al., 2014a,b; Demirtaş et al., 2019). In addition, most BNMs are optimized to fit the high-level statistics such as FC instead of raw BOLD timeseries (Deco et al., 2013a,b; Wang et al., 2019a), which may not capture the dynamic features of fMRI signals accurately.

Thankfully, substantial progress has been made in term of parameter estimation for BNMs in recent years. Using expectation-maximization (EM) approach, Wang et al. (2019a) estimated a total of 138 parameters of a large-scale dynamic mean-field model including region-specific recurrent excitation strength and subcortical input level, though only one global scaling constant was estimated for all inter-regional connections. Besides, a new SC-guided computational approach to estimate whole-brain EC has been proposed and applied to language development (Hahn et al., 2019). This new approach uses SC as initial guess for EC which is iteratively updated according to a gradient descent algorithm to maximize the similarity between modeled FC and empirical FC. Lastly, a novel variant of BNM has been developed recently that is capable of individual EC inference in whole-brain network (Gilson et al., 2016, 2018, 2020). Different from traditional BNMs, this new framework models local neuronal dynamics with the multivariate Ornstein-Uhlenbeck (MOU) process and estimates EC by maximum likelihood, which is referred to as MOU-EC. From a dynamic systems point of view, MOU-EC corresponds to a network with linear feedback which is equivalent to the multivariate autoregressive (MAR) process, or the linearization of the non-linear Wilson-Cowan neuronal model (Wilson and Cowan, 1972; Gilson et al., 2020). Characterized by a balance between tractability and rich dynamics on parameter variations, MOU-EC offers a comprehensive new set of tools to study distributed cognition and neuropathology (Gilson et al., 2020). Nevertheless, the MOU-EC approach does not model within-region excitatory and inhibitory interactions.

In summary, BNM and DCM share similarities but also bear significant differences. At the common side, they both have a generative model for neuronal dynamics and a measurement model to convert the neuronal activity to BOLD signal, and usually integrate an optimization scheme to estimate EC. At the different note, DCM focuses on estimating individual EC at single subject level, while BNM focuses on simulating fMRI data. Based on such distinct design objectives, DCM utilizes a relatively simple bilinear neural model while BNM employs more realistic spiking or neural mass models. Also, BNM usually utilizes SC as a proxy for synaptic efficacy or a backbone for EC, while DCM does not necessarily require structural information, though it can be used as a prior constraint (Sokolov et al., 2020). Besides, DCM is usually confined to small networks (up to tens of nodes) while BNM applies to large-scale whole brain networks (up to hundreds of nodes). Moreover, DCM uses a Bayesian framework to estimate EC which provides the full posterior probability distributions of model parameters and enables Bayesian model comparison. In contrast, the optimization schemes in BNMs are diverse and generally provide point estimates of model parameters, which do not possess the capability of automatic pruning of connections. Lastly, DCM generally fits to raw BOLD timeseries (but see spectral and regression DCM) while BNM usually fits to high-level summary statistics such as FC. It should be recognized that the latest developments of DCM for fMRI (e.g., NMM-based DCM, regression DCM) and BNM (e.g., MOU-EC) have strived to overcome the limitations of each framework, representing a convergence between DCM and BNM (Stephan et al., 2015).

### The neural model

#### Spiking neuron model

For the “direct simulation” approach in BNM, individual neurons are commonly described by the classical integrate-and-fire (IF) spiking neurons (Tuckwell, 1988) connected with biophysical synapses. The membrane potential (*V*_m_) is governed by the following equation:


(7) $$C_{\text{m}}\,\frac{d\,V_{\text{m}}}{d\,t}=-g_{\text{m}}\,\left(V_{\text{m}}-V_{\text{L}}\right)-I_{\text{syn}}+I_{\text{Ext}}$$

where *C*_m_ is the membrane capacitance, *g*_m_ the leak conductance, *V*_L_ the resting potential, and *I*_*Ext*_ the external input. When *V*_m_ crosses a threshold *V*_th_ in the upward direction, a spike is generated and the membrane potential is reset to a value *V*_res_ for a refractory time period τ_res_. The total synaptic current *I*_*syn*_ is a summation of recurrent excitatory/inhibitory inputs from local region and excitatory inputs from other brain areas; Excitatory inputs are mediated by both AMPA and NMDA receptors, while inhibitory inputs are mediated by GABA_A_ receptors. Synaptic currents follow the following biophysical model (Deco and Jirsa, 2012; Deco et al., 2014b):


(8) $$I_{\text{syn}}\,\left(t\right)=W\,g_{\text{syn}}\,B\,\left(V_{\text{m}}\right)\,s\,\left(V_{\text{m}}-V_{\text{syn}}\right)$$

where *W* is the synaptic weight, *g*_syn_ the maximal synaptic conductance, *s* the gating variable, and *V*_syn_ is the synaptic reversal potential. The magnesium block function *B*(*V*_m_) = 1/(1 + exp(−0.062*V*_m_)/3.57) for NMDA currents and *B*(*V*_m_) = 1 for AMPA and GABA_*A*_ currents. The gating variable *s* follows the following dynamics:

For AMPA or GABA_*A*_ current:


(9) $$\frac{d\,s}{d\,t}=\frac{-s}{\tau_{A\,M\,P\,A/G\,A\,B\,A}}+\sum_{\text{i}}\delta \,\left(t-t_{\text{i}}\right)$$

For NMDA current:


(10) $$\frac{d\,s}{d\,t}=\frac{-s}{\tau_{N\,M\,D\,A}}+\alpha_{s}\,x\,\left(1-s\right)$$

(11) $$\frac{d\,x}{d\,t}=\frac{-x}{\tau_{x}}+\sum_{\text{i}}\delta \,\left(t-t_{\text{i}}\right)$$

where *x* models the neurotransmitter concentration with the rise time constant τ_*x*_. τ_*AMPA*_, τ_*GABA*_ and τ_*NMDA*_ are the decay time constants for AMPA, GABAa and NMDA synapses, respectively, *t*_i_ is the presynaptic spike times, and α_*s*_ controls the saturation properties of NMDA.

#### Dynamic mean field model

The dynamic mean field model (MFM) has been frequently used in BNMs (Deco et al., 2013b, 2014a,b; Wang et al., 2019a), which is derived by mean-field reduction of the spiking neuronal network model. Each brain region is described by the following set of non-linear stochastic differential equations (Deco et al., 2013b; Wang et al., 2019a):


(12) $$\frac{{\mathrm{dS}}_{i}}{\mathrm{dt}}=-\frac{S_{i}}{\tau_{s}}+r\,\left(1-S_{i}\right)\,H\,\left(x_{i}\right)+\sigma \,v_{i}$$

(13) $$H\,\left(x_{\text{i}}\right)=\frac{a\,x_{\text{i}}-b}{1-\text{exp}\,\left(-d\,\left(a\,x_{\text{i}}-b\right)\right)}$$

(14) $$x_{\text{i}}=w\,J\,S_{\text{i}}+G\,J\,\sum_{j}C_{i\,j}\,S_{j}+I$$

where *S*_i_, *x*_i_ and *H*(*x*_i_) represent the average synaptic gating variable, the total input current, and the population firing rate at brain region *i*, respectively. *G* is a global scaling factor, *J* is the value of synaptic coupling, *w* is the recurrent connection strength, and *C*_*ij*_ denotes the SC between region *i* and region *j*. τ_*s*_ and *r* are kinetic parameters, and *a*, *b* and *d* are parameters of the function *H*(*x*_i_). *v*_i_ represents uncorrelated Gaussian noise with standard deviation σ. The simulated neural (synaptic) activity *S*_i_ is fed to the same hemodynamic model as DCM (Eqn. 2–6) to generate simulated BOLD timeseries.

### Development and implementation of biophysical network models

Development of BNMs includes the following major steps: **(1)** parcellating the brain into discrete regions; **(2)** extracting whole-brain fMRI-BOLD timeseries and calculating FC; **(3)** computing SC based on diffusion MRI data; **(4)** representing each network node with populations of spiking neurons or neural-field/NMM of local neuronal populations; **(5)** linking individual network nodes with long-range connections based on SC; **(6)** transforming the network activities to simulated BOLD signals *via* a hemodynamic model; and **(7)** fitting model parameters to FC *via* an optimization scheme.

There are a number of computational software packages to facilitate the development and implementation of BNMs including NEURON (Carnevale and Hines, 2006), BRIAN (Goodman and Brette, 2009; Stimberg et al., 2019), NEST (Gewaltig and Diesmann, 2007), and The Virtual Brain (TVB; Jirsa et al., 2010; Sanz-Leon et al., 2013). Specifically, NEURON is the preferred simulation environment for the construction of morphologically and biophysically realistic neuronal models and networks. BRAIN and NEST are tailored for spiking network models that focus on the dynamics and structures of neural systems rather than the exact morphology and physiology of individual neurons. Different from NEURON, BRAIN and NEST that concentrate on simulation of individual neurons within small brain regions, TVB is a platform specifically designed for constructing and simulating personalized brain networks based on multimodal neuroimaging data such as fMRI, diffusion MRI, MEG and EEG. Conveniently, one could choose different neural mass or neural field models of local dynamics from TVB’s predefined model classes and apply different measures of anatomical connectivity [CoCoMac or human diffusion-weighted imaging (DWI) data].

### Application to cognitive neuroscience

One fundamental question in cognitive neuroscience is how different cognitive states such as attention, sleep and wakefulness are defined mechanistically and switch from one state to the other. Existing definitions focus on resting networks and statistical description of functional activity patterns (Barttfeld et al., 2015; Tagliazucchi et al., 2016), which do not provide a mechanistic understanding of the dynamical coordination between neural systems. Recent breakthrough from computational neuroscience has filled this important gap (Deco et al., 2019; Kringelbach and Deco, 2020). Based on the concept of metastabilty (i.e., the ability of a system to maintain its equilibrium for an extended time in the presence of small perturbations), Deco et al. (2019) defined brain state as an ensemble of “metastable substates” each characterized by a probabilistic stability and occurrence frequency. This novel definition allows for systematic investigation of brain state transition. Using a unique fMRI and EEG dataset recorded from healthy subjects during awake and sleep conditions, Deco et al. (2019) fit a whole-brain generative BNM to the probabilistic metastable substates (PMS) space of the empirical data corresponding to the awake and sleep conditions. It was demonstrated that *in silico* stimulation predicted by the BNM can accurately force transitions between different brain states, and in particular, from deep sleep to wakefulness and vice versa. These findings provide valuable insights how and where to induce a brain state transition using whole-brain BNM, which may potentially apply to restore the pathological brain state to normal state using external stimulation. The new brain state definition and modeling framework were recently applied to study the effect of external stimulation on functional networks in the aging healthy human brain (Escrichs et al., 2022). The authors first characterized the brain states as PMS space in two groups of subjects [middle-aged adults (age < 65) and older adults (age ≥ 65)], based on a large-cohort resting-state fMRI dataset (*N* = 310 for each group). A whole-brain BNM was then developed and fit to the PMS and *in silico* stimulation with region-specific intensity was applied to induce transitions from the brain states of the older group to those of the middle-age group. The authors discovered that the precuneus, a brain region belonging to DMN and involved in a variety of complex functions such as episodic memory and visuospatial processing, is the best stimulation target for brain state transition. This elegant study suggests that generative models for neuroimaging data could potentially serve as gateway for the design of novel brain perturbation techniques to reverse the adverse effects of aging on cognitive functions.

Another important contribution of BNM to cognitive neuroscience is the manifestation of the dynamical origin of slowness of thought during task-based cognition (Kringelbach et al., 2015). This discovery relies on a major finding from whole-brain BNMs that the brain is not only metastable (Tognoli and Kelso, 2014), but also maximally metastable (Cabral et al., 2014; Kringelbach et al., 2015). Such dynamical property has far-reaching implications as it leads to a characteristic slowness of spontaneous dynamics when the brain network enters the state of transition, a phenomenon termed “critical slowing down” (Kringelbach et al., 2015; Meisel et al., 2015). Notably, a previous whole-brain BNM has also revealed a critical slowing down on the edge of a criticality (Deco et al., 2013b). Together these modeling studies suggest that optimal task processing requires extensive examination of the dynamical repositories of brain networks, which explains the nature of slowness of cognition. It should be stressed that such fundamental insights can only be made possible by generative neuronal modeling (Deco and Jirsa, 2012) because traditional FC analysis cannot reveal whether the brain is maximally metastable or not.

Since its introduction, the MOU-EC model (Gilson et al., 2016) has been applied to offer insights into the neural mechanisms of cognition. It has been well documented that perceptual categorization, the mapping of sensory stimuli to category labels, involves a two-stage processing hierarchy in both the visual and auditory systems (Riesenhuber and Poggio, 2000; Ashby and Spiering, 2004; Jiang et al., 2007, 2018). The first stage is a “bottom-up” stage where neurons in the sensory cortices learn to respond to stimulus features while the second stage is a “top-down” stage where neurons in higher cortical areas learn to classify the stimulus-selective inputs from the first stage. To investigate whether the two-stage processing hierarchy also exists in the somatosensory system, Malone et al. (2019) designed an experiment where human participants, after training to label vibrotactile stimuli presented to their right forearm, underwent an fMRI scan while actively engaging in categorizing the stimuli. The authors first applied representational similarity analysis to identify the stimulus- and category- selective areas. Next they utilized the MOU-EC method (Gilson et al., 2016) to estimate whole-brain EC among 200 regions. It was observed that the influence (i.e., effective drive) from most of the category-selective areas to the stimulus-selective areas was much higher than that in the opposite direction. These findings support the two-stage processing hierarchy in the somatosensory system, providing a unified computational principle for perceptual categorization. The MOU-EC model has also been used to study the neural mechanisms underlying the engagement of functionally specialized brain regions, and in particular, how brain network connectivity is modulated under different cognitive conditions to give rise to differential regional dynamics (Gilson et al., 2018).

## Dynamic Neural Model with direct parameterization

### Overview

In addition to DCM and BNM, the two major types of generative models for fMRI, there exist other modeling frameworks that estimate EC and study connectome in a generative fashion (Havlicek et al., 2011; Olier et al., 2013; Fukushima et al., 2015; Becker et al., 2018; Singh et al., 2020; Li et al., 2021). One framework that shows promising application to cognitive and clinical neuroscience is Dynamic Neural Model (DNM) with direct parameterization. This type of modeling approach attempts to balance the complexity of BNM with the identifiability of DCM so that the model is both sufficiently realistic and equipped with the capability to efficiently estimate individual connections at single subject level. Notably, DNM utilizes non-linear dynamic models or NMMs to model local neuronal population activity, thus able to capture the extended patterns of the spatiotemporal dynamics of brain networks.

One recent development of DNM is termed Mesoscale Individualized Neurodynamic (MINDy) modeling that fits non-linear dynamical neural models directly to fMRI data (Singh et al., 2020). To achieve computational efficiency, MINDy models regional neural population activity in the fMRI scale (instead of neuronal firing scale) using an abstracted NMM. After converting the continuous-time neural model to a discrete-time analog, an objective function is formed by summing up the one-step prediction errors of empirical BOLD timeseries, which allows the calculation of error gradients analytically. Using a computationally efficient optimization algorithm [Nesterov-Accelerated Adaptive Moment Estimation (NADAM, Dozat, 2016)], model parameters are estimated to minimize the objective function. The high computational efficiency enables identification of EC parameters in large-scale networks with hundreds of nodes in just 1–3 min per subject, making it ideally suitable for big dataset application. Notably, MINDy requires no prior anatomical constraints or long-term summary statistics for model inversion and provides estimation of individual connection parameters in an individualized and efficient manner, representing a significant departure from existing methods.

**FIGURE 3:**
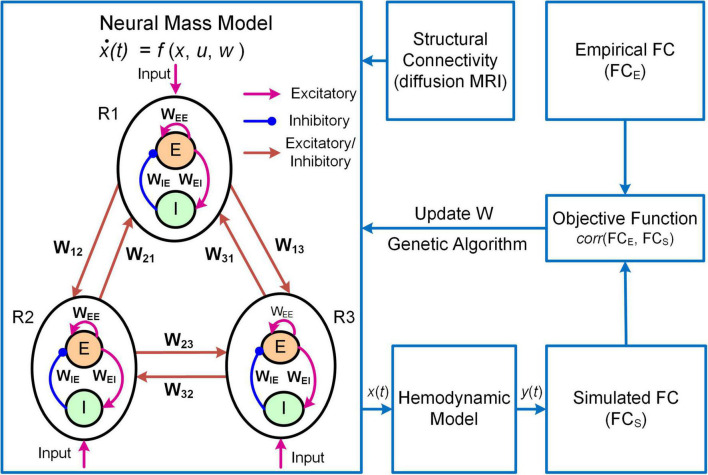
Overview of the MNMI framework. The neural activity [*x*(*t*)] is generated by the Wilson-Cowan network model (Wilson and Cowan, 1972) consisting of multiple brain regions (R1, R2, etc.). Each region contains one excitatory (*E*) and one inhibitory (*I*) neural populations coupled with reciprocal connections and receives spontaneous input (*u*). Different brain regions are connected *via* long-range fibers whose baseline strengths are determined by structural connectivity from diffusion MRI. The regional neural activity is converted to corresponding BOLD signal [*y*(*t*)] *via* a hemodynamic model (Friston et al., 2003). Both intra-regional recurrent excitation (*W*_*EE*_) and inhibition (*W*_*IE*_) weights and inter-regional connection strengths (*W*_12_, *W*_21_, etc.) as well as spontaneous input (*u*) are estimated using genetic algorithm to maximize the similarity between simulated and empirical FC. Adapted from Li et al. (2021) under the Creative Commons Attribution License (CC BY).

Another example of DNM with direct parameterization is a framework termed Multiscale Neural Model Inversion (MNMI, Figure 3; Li et al., 2021). In MNMI, the neuronal activity is generated by a neural mass network model comprised of multiple brain regions. Each region contains one excitatory and one inhibitory neural populations coupled with reciprocal connections, and the intrinsic dynamics are described by the classical Wilson-Cowan model (Wilson and Cowan, 1972). The excitatory neural populations within different brain regions are connected *via* long-range fibers whose baseline connection strengths are determined by SC from diffusion MRI; weak inter-regional connections are removed to construct sparse networks and avoid the problem of over-parameterization. The regional neural activity is converted to corresponding BOLD signal *via* a hemodynamic model (Friston et al., 2003) and FC is computed using Pearson’s correlation. MNMI then applies genetic algorithm, a biologically inspired optimization algorithm, to estimate both intra-regional and inter-regional coupling strengths to minimize the difference between simulated and empirical FC. Similar to MINDy, MNMI estimates EC for individual connections at single subject level, thus offering personalized assessment. MNMI differs from MINDy in several aspects including using a more biologically informed NMM with excitatory and inhibitory interactions, modeling neural activity in neuronal firing scale (vs. fMRI scale), utilizing structural information to constrain EC estimation, and applying genetic algorithm (vs. gradient descent algorithm) to estimate model parameters. One limitation of MNMI is its heavy computational burden, which prevents large-scale whole-brain network applications. Nevertheless, MNMI provides a multiscale modeling framework to fathom deeper brain connectivity features in health and disease.

### The neural model

Neural mass models are commonly used to model local neuronal dynamics in DNM. For instance, MINDy and MNMI use one-state and two-state NMMs, respectively. The NMM in MNMI is described by the following differential equations (Wilson and Cowan, 1972; Li et al., 2021):


(15) $$\tau_{e}\,\frac{d\,E_{j}\,\left(t\right)}{d\,t}=-E_{j}\,\left(t\right)+S\,\left(\sum_{\text{k}}W_{k\,j}\,C_{k\,j}\,E_{\text{k}}\,\left(t\right)+W_{E\,E}^{\text{j}}\,E_{\text{j}}\,\left(t\right)-W_{I\,E}^{\text{j}}\,I_{\text{j}}\,\left(t\right)+u+\varepsilon \,\left(t\right)\right)$$

(16) $$\tau_{\text{i}}\,\frac{d\,I_{\text{j}}\,\left(t\right)}{d\,t}=-I_{\text{j}}\,\left(t\right)+S\,\left(W_{E\,I}^{\text{j}}\,E_{\text{j}}\,\left(t\right)+\varepsilon \,\left(t\right)\right)$$

where *E*_j_ and *I*_j_ are the mean firing rates of excitatory and inhibitory neural populations in brain region *j*, τ_*e*_ and τ_i_ are the excitatory and inhibitory time constants, $W_{E\,E}^{\text{j}}$, $W_{E\,I}^{\text{j}}$ and $W_{I\,E}^{\text{j}}$ are the local coupling strengths from excitatory to excitatory neural population, from excitatory to inhibitory neural population and from inhibitory to excitatory neural population, respectively. The variable *u* is a constant external input representing average extrinsic drive from other un-modeled brain regions, and ε(*t*) is random additive noise following a normal distribution. The long-range connectivity strength from region *k* to region *j* is represented by *W*_kj_ scaled by empirical SC (*C*_kj_). The non-linear response function *S* is modeled as a sigmoid function $S=1/\left(1+e^{-\left(\frac{x-\mu }{\sigma }\right)}\right)$. MNMI estimates connection parameters *W*_kj_, $W_{\text{EE}}^{\text{j}}$, $W_{\text{IE}}^{\text{j}}$, and input *u* based on individual SC and FC.

### Development and implementation of Dynamic Neural Models

The development steps of DNMs are generally similar to BNMs: **(1)** parcellating the brain into discrete regions; **(2)** selecting brain regions to model and extracting fMRI-BOLD timeseries; **(3)** calculating FC and/or SC if necessary; **(4)** representing each network node with a NMM of local neuronal populations; **(5)** linking individual network nodes with long-range connections with or without SC constraint; **(6)** deconvolving the empirical BOLD signals with a hemodynamic response function (HRF, Singh et al., 2020) or transforming the network activities to simulated BOLD signals *via* a hemodynamic model (Li et al., 2021); and **(7)** fitting model parameters to deconvolved BOLD signals or FC *via* an optimization scheme. Due to the flexibility in neuronal model, SC utilization, choice of objective function and optimization algorithm, DNMs are generally implemented using customized scripts with MATLAB or other computing languages (e.g., C++).

### Application to clinical neuroscience

DNM has been applied to study the circuit mechanisms of major depressive disorder (MDD). MDD is a leading cause of chronic disability worldwide with a lifetime prevalence of up to 17% (Kessler et al., 2005), but the underlying pathophysiological mechanisms remain elusive. Functional connectome analysis indicates that MDD can be characterized as a disorder with dysfunctional connectivity and regulation among multiple resting-state networks including the DMN, salience network, executive control network and limbic network (Menon, 2011; Dutta et al., 2014; Mulders et al., 2015; Drysdale et al., 2017). However, two important questions remain unresolved. First, it is not clear which functional networks play a central role and which functional networks play a subordinate role in MDD pathogenesis. Second, it is unclear whether the dysconnectivity or dysregulation originates from limbic or cortical system and whether such dysregulation results from intrinsic (intra-regional) or extrinsic (inter-regional) mechanisms. Answering these two questions is not only important for deeper mechanistic understanding of MDD pathology but also necessary for more targeted treatments.

**FIGURE 4:**
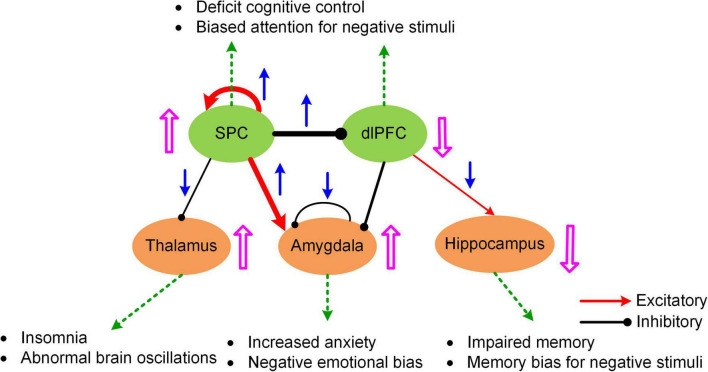
A hypothetic model of executive-limbic malfunction in MDD. MDD is mediated by increased recurrent excitation in the superior parietal cortex (SPC) and greater inhibition from the SPC to the dorsolateral prefrontal cortex (dlPFC), leading to increased SPC activity and decreased dlPFC response, which may underlie deficit cognitive control and biased attention for negative stimuli. In addition, the excitatory drive from the SPC to the amygdala is abnormally elevated in MDD. Combined with reduced recurrent inhibition, the amygdala shows hyperactivity which causes increased anxiety and biased processing of negative stimuli. Besides, the inhibitory drive from the SPC to the thalamus is reduced while the excitatory projection from the dlPFC to the hippocampus is abnormally decreased in MDD. The former change could result in abnormal brain oscillations and insomnia while the latter change could account for impaired memory function and biased memory for negative stimuli. The blue arrows indicate the change of the connection strengths in MDD from normal control. The pink UP/DOWN arrows next to the brain regions indicate the change in neural responses in MDD compared to normal control. Adapted from Li et al. (2021) under the Creative Commons Attribution License (CC BY).

The MNMI framework is well suited to address these questions due to its biological realism utilizing a physiologically informed NMM and its multiscale nature incorporating both intra-regional and inter-regional neural interactions. By applying the MNMI framework to a large sample-size resting-state fMRI dataset consisting of 100 MDDs and 100 normal control (NC) healthy subjects, Li et al. (2021) demonstrated that MDD pathology is more likely caused by aberrant circuit interactions and dynamics within a core “executive-limbic” network rather than the “default mode-salience” network, consistent with the long-standing hypothesis of limbic-cortical dysregulation in MDD (Mayberg, 1997, 2002, 2003; Davidson et al., 2002; Disner et al., 2011). Notably, model results indicated that both limbic and cortical systems and both intra-regional and inter-regional connectivities could play a role in MDD pathology. Specifically, MNMI analysis showed that recurrent inhibition within the amygdala was abnormally decreased and the excitatory EC from the superior parietal cortex (SPC) to the amygdala was abnormally increased, which may underlie hyperactivity of the amygdala in MDD (Drevets, 2001; Siegle et al., 2002), leading to increased anxiety and cognitive bias over negative stimuli (Figure 4; Disner et al., 2011). In addition, the EC from the SPC to the dorsolateral prefrontal cortex (dlPFC) switched from excitation in NC to inhibition in MDD, which well explains dlPFC hypoactivity (Fales et al., 2008; Hamilton et al., 2012), resulting in deficit cognitive control (Figure 4; Disner et al., 2011). The model also revealed other abnormal connectivity patterns in MDD including elevated recurrent excitation in the SPC, reduced SPC inhibition on the thalamus and decreased dlPFC excitation on the hippocampus, which may underlie biased attention for negative stimuli, abnormal brain oscillations and impaired memory function, respectively (Figure 4; Disner et al., 2011; Li et al., 2021). Overall, by employing a biologically plausible NMM, the MNMI framework provides a mechanistic account of circuit dysfunction in MDD which highlights the importance of targeting the executive-limbic system for maximal therapeutic benefits.

## Summary and future direction

Driven by the rapid advances in non-invasive neuroimaging techniques, the young emerging field of human connectomics has made significant accomplishments in characterizing the large-scale organizational features of both structural and functional brain networks. Notwithstanding, the potential of connectomics to answer fundamental neuroscience and clinical questions has yet to bring into full play. To achieve such important goal, computational connectomics has moved beyond the anatomical and statistical description of connectivity to more mechanistic formulation of the neural processes underlying neuroimaging data (i.e., mechanistic connectome). Mechanistic connectome based on generative modeling of fMRI offers a natural and principled tool to link microscopic or mesoscopic neural process with macroscopic BOLD dynamics, which enables mechanistic understanding of brain cognitive functions in heath and dysfunction in diseases. It is important to note that, unlike static structural connectome, mechanistic connectome based on effective connectivity is parameterized by the state of the brain. That is, one would obtain a very different mechanistic connectome when the task demands change, or when the stimuli challenge, or when endogenous activity switches to a different state (e.g., inward vs. outwardly directed attention) (Friston et al., 2003; Jung et al., 2018; Park et al., 2021). Thus, there will not be a conventional atlas for the mechanistic connectome like one could obtain for the structural connectome.

Recent years have seen tremendous development and expansion of generative model-based connectome analysis toolsets. Two well-established and widely used modeling frameworks include DCM and BNM, which represent two approaches at the opposite end of biological realism and estimation tractability. Specifically, while DCM allows estimation of full connection parameters at individual subject level, the physiological interpretability for model parameters is limited due to the abstract bilinear state model. On the other hand, though BNM incorporates more physiologically grounded neuronal models for fMRI generation, its identifiability is limited to one or a small subset of parameters often at the group-average level. Effort to combine the advantages of DCM with BNM has led to the development of a different type of modeling framework that can be categorized as Dynamic Neural Model (DNM) with direct parameterization. DNM seeks to model population-level neuronal dynamics accurately with biophysically plausible NMMs and estimate physiologically meaningful parameters for individual connections at single subject level. It should be noted that the boundary among these three types of generative models is diminishing with the latest developments of DCM and BNM which utilize more biophysically informed models and are equipped with the capability for efficient EC estimation of large-scale networks at both individual connection and individual subject levels. One should expect the convergence of DCM, BNM and DNM continues in the future.

**FIGURE 5:**
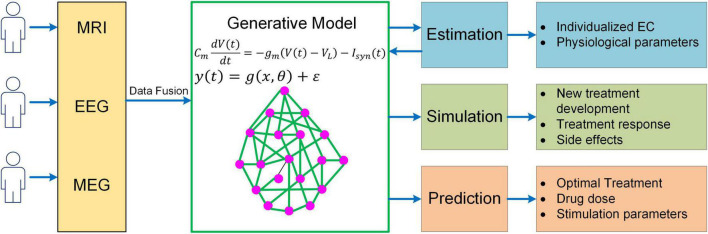
A unified mechanistic pipeline for generative model-based neuroimaging analysis to treat brain disorders. In this analysis pipeline, individual subjects first undergo multiple neuroimaging scans/recordings such as MRI, EEG and MEG. The multi-modal neuroimaging data are then combined with data fusion and fed into the generative model to estimate individualized EC and other relevant physiological parameters such as neuromodulatory levels. The estimated model parameters are then fed back into the generative model to simulate existing treatment response and new treatment development as well as their side effects. Based on the simulation outcome, the generative model will predict optimal treatment strategy for the patient along with drug dose or stimulation parameters.

While much progress has been made, more needs to be done to meet the challenges in neuroscience. It should be recognized that even the most sophisticated BNM is only a highly simplified representation of the human brain, yet more complex models would make parameter inference much more difficult, raising the question of how to determine the right level of complexity in generative modeling. One important rule of thumb is that models should only be considered that are in the right ballpark of complexity to address the question at hand. That is, they need to have parameters relating to the quantities of interest (interpretability), while not being more complex than the data can accommodate (given the limited resolution of fMRI). This principle has been well implemented in DCM *via* Bayesian model selection, a process where different candidates of models are iteratively generated and compared to reach the models that have the optimal level of complexity (Stephan et al., 2009; Rosa et al., 2012). Specifically, the optimal model optimizes the trade-off between accuracy and complexity, which is quantified by the log model evidence (i.e., log p(y|m); Zeidman et al., 2019a,b). The topic of complexity deserves more consideration in future generative modeling studies given the need to explore more physiologically based neuroscience questions (e.g., neuromodulatory effects on cognitive functions). Also, to have a thorough understanding of the neural mechanisms of cognition, a truly multiscale model is wanted which has the capability to link cellular, circuit, network and system dynamics with behavioral response. Moreover, to enable more accurate estimation of model parameters, generative models need to integrate multimodal neuroimaging data (e.g., fMRI, MEG, and EEG) into a unified framework. Lastly, in order to apply to clinical interventions, generative models should be able to explore new treatment paradigms such as non-invasive brain stimulation for brain disorders, predict optimal personalized treatment and simulate the treatment outcome (Figure 5). Addressing such grand challenges will lead to a new class of generative models for neuroimaging data that not only revolutionizes the field of human connectomics but also significantly advances our understanding of the human brain and neuropsychiatric disorders.

## Author contributions

GL and P-TY contributed to the conception of the work, revised the manuscript, and approved the final version of the manuscript. GL drafted the manuscript. Both authors contributed to the article and approved the submitted version.

## Footnotes

1
https://www.fil.ion.ucl.ac.uk/spm/software/
